# Synergistic Antimicrobial Effects of Citric Acid-Based Deep Eutectic Bioactive Agents in Chitosan Coatings for Refrigerated Shrimp Preservation

**DOI:** 10.3390/foods15091601

**Published:** 2026-05-06

**Authors:** Guoxing Ruan, Ziru Dai, Jawad Ashraf, Faisal Hayat, Yu Wang, Yuansen Liu, Ping Shi, Weibing Lan, Tingcai Pang, Hafiz Umer Javed

**Affiliations:** 1 Laboratory of Beibu Gulf Marine Product Healthy Aquaculture and Green Processing, Guangxi Zhuang Autonomous Region Engineering Research Center of Marine Food Nutrition and Processing Technology Innovation, College of Food Engineering, Beibu Gulf University, Qinzhou 531011, China; 2 Guangxi College and University Key Laboratory of High-Value Utilization of Seafood and Prepared Food in Beibu Gulf, Qinzhou 531011, China; 3 College of Food Science and Engineering, Bohai University, Jinzhou 121013, China; 4 Department of Integrative Agriculture, College of Agriculture and Veterinary Medicine, United Arab Emirates University, Al Ain P.O. Box 15551, United Arab Emirates

**Keywords:** antibacterial activity, chitosan coating, deep eutectic agent, shrimp preservation

## Abstract

*Penaeuschinensis* is an economically important seafood species valued for its nutritional quality and global market demand. However, its high perishability makes it highly susceptible to rapid quality deterioration during refrigerated storage, primarily due to microbial proliferation, enzymatic activity, and oxidative reactions. To address these challenges, this study proposes a sustainable chitosan-based coating incorporating a citric acid–choline chloride deep eutectic agent (CA-DEA) as an innovative preservation strategy for shrimp. The composite coating demonstrated markedly enhanced antioxidant and antibacterial activities compared to CTS or CA-DEA alone. The CTS-CA-DEA coating effectively preserved shrimp quality over 8 days of refrigerated storage, as evidenced by reduced discoloration, moisture loss, and textural degradation during storage. These quality improvements were accompanied by greater stability of key biochemical indicators, including peroxide value, pH, total volatile basic nitrogen, and protein content, indicating a slower progression of spoilage reactions. Electronic nose analysis further revealed a reduced generation of lipid- and protein-derived volatile compounds associated with shrimp deterioration, consistent with the observed physicochemical changes. Based on the accepted TVB-N acceptability threshold (30–35 mg/100 g), the CTS-CA-DEA treatment prolonged the estimated acceptable refrigerated storage period to approximately 7 days, compared with only about 4 days for the uncoated control, clearly demonstrating the beneficial effect of the composite antimicrobial coating. Collectively, these results demonstrate that the CTS-CA-DEA coating is an eco-friendly preservation strategy that integrates barrier protection, antimicrobial activity, and antioxidant defense, thereby extending refrigerated shelf life while maintaining shrimp quality.

## 1. Introduction

Shrimp are highly valued globally for their appealing texture, rich protein content, and abundance of polyunsaturated fatty acids; however, they remain among the most perishable aquatic food products [1,2]. Their rapid postharvest deterioration is primarily attributed to high water activity, near-neutral pH, and the activity of endogenous enzymes, which render shrimp highly susceptible to microbial proliferation and protein-lipid oxidation [3]. These spoilage reactions result in melanosis, texture softening, off-odor formation, and substantial reductions in shelf life [4]. Although traditional preservation methods such as salting, drying, fermentation, chilling, and freezing are widely applied in food processing, their effectiveness in maintaining the quality of highly perishable shrimp products remains limited [5]. Moreover, the use of chemical preservatives such as sulfites and nitrates has raised growing regulatory restrictions and consumer health concerns [6]. Consequently, the development of safe, environmentally friendly, and multifunctional preservation strategies capable of targeting the specific spoilage mechanisms of shrimp has become an important research focus for the seafood industry.

To address these challenges, natural organic acids, including lactic, acetic, and citric acid, have been widely investigated as safer and more environmentally friendly alternatives to synthetic preservatives. However, their antimicrobial efficacy at food-permissible concentrations is often insufficient to effectively control the rapid biochemical and microbiological changes characteristic of aquatic products [5]. This limitation has encouraged researchers to explore green materials such as deep eutectic solvents (DESs), which are formed through hydrogen-bond interactions between hydrogen-bond donors and acceptors and exhibit favorable properties including biodegradability and tunable physicochemical behavior [7,8]. More recently, DESs have attracted attention as potential preservation agents because their antimicrobial and antioxidant activities can be adjusted through component selection [9]. Although DESs can be formed from various components such as organic acids, sugars, amino acids, and quaternary ammonium salts [10], the present study focuses on the citric acid–choline chloride (CA-ChCl) system due to its food compatibility and potential application in seafood preservation. Citric acid (CA), a naturally occurring polycarboxylic acid, readily forms eutectic structures with choline chloride (ChCl), and CA-ChCl DEAs have been reported to exhibit intrinsic antibacterial and antioxidative properties [9]. Furthermore, CA has independently been shown to reduce TVB-N accumulation and improve sensory attributes in rainbow trout stored in slurry ice [11] and to act synergistically with rosemary extract in suppressing microbial growth and lipid oxidation in chilled Pacific white shrimp [12]. However, the synergistic integration of CA-based deep eutectic agents with chitosan coatings for controlling multiple spoilage pathways in shrimp has not yet been systematically investigated. These findings highlight the promising preservation potential of CA-based eutectic systems and support the development of environmentally friendly and multifunctional preservation materials for shrimp.

Chitosan (CTS), a naturally derived polysaccharide obtained from crustacean shells, is widely recognized for its intrinsic antimicrobial activity, excellent film-forming ability, and biodegradability, making it a promising material for seafood preservation applications [13]. Extensive research has shown that CTS-based coatings can modulate the microenvironment surrounding seafood products by limiting oxygen diffusion, controlling moisture transfer, delaying melanosis development, suppressing microbial proliferation, and mitigating oxidative deterioration, thereby helping to stabilize texture integrity and overall quality during storage [14]. The functional performance of CTS systems can be further enhanced by incorporating bioactive additives; for example, chitosan coatings enriched with natural antioxidants such as tea polyphenols have been reported to inhibit microbial growth and retard lipid oxidation in shrimp during refrigerated storage [15]. Nevertheless, the preservation efficacy of CTS coatings largely depends on the characteristics and compatibility of the functional agents incorporated into the polymer matrix. In this context, the incorporation of citric acid-based deep eutectic agents (CA-DEAs) represents a promising approach, as CA-DEAs possess intrinsic antimicrobial and antioxidant properties while also improving intermolecular interactions and structural stability within the polymer network. Therefore, integrating CTS with a bioactive CA-DEA system provides a rational and targeted strategy for developing an advanced multifunctional preservation coating capable of addressing the rapid spoilage mechanisms of shrimp.

Based on these considerations, we hypothesize that incorporating CA-DEA into CTS coatings would generate a synergistic bio-preservation system capable of more effectively controlling the microbial, enzymatic, and oxidative deterioration of shrimp during refrigerated storage. Specifically, the CA-DEA system with a 4:1 molar ratio, selected based on previous reports and preliminary optimization showing superior antioxidant and antibacterial performance, may enhance the functional stability of the coating, strengthen intermolecular interactions within the CTS structure (as supported by FTIR analysis), and provide targeted inhibition of melanosis development, lipid oxidation, and volatile nitrogen formation. To evaluate this hypothesis, and to address the limited research on the combined application of CA-based deep eutectic agents and chitosan coatings for shrimp preservation, the present study aimed to: (i) synthesize CA-DEAs at the established optimal molar ratio and evaluate different incorporation concentrations to identify the most effective level for the CTS coating formulation; (ii) develop CA-DEA-plasticized CTS coatings and examine their structural characteristics and functional properties; and (iii) systematically assess the effects of these coatings on microbial quality, physicochemical attributes, oxidative stability, and sensory-related quality changes in shrimp during refrigerated storage. By integrating antioxidant, antibacterial, and barrier functions within a single coating system, this study provides a targeted preservation strategy specifically designed to mitigate the rapid spoilage mechanisms of shrimp. This work seeks to establish CTS-CA-DEA coatings as a green, multifunctional, and seafood-specific preservation technology with strong potential to enhance the postharvest stability of shrimp.

## 2. Materials and Methods

### 2.1. Materials and Reagents

Chitosan (degree of deacetylation ≥90%, viscosity 100–200 mPa s), choline chloride (ChCl, AR, 98%), citric acid (CA, AR, 99.5%), DPPH (2,2-diphenyl-1-picrylhydrazyl, AR, 98%), and ABTS (2,2′-azino-bis(3-ethylbenzothiazoline-6-sulfonic acid, AR, 98%) were purchased from Aladdin (Shanghai, China). Acetic acid, methyl red, and bromocresol green (all analytical grade) were obtained from Sinopharm Chemical Reagent Co., Ltd. (Beijing, China). Ferric chloride, boric acid, magnesium chloride, and ammonium thiocyanate (AR grade) were supplied by Tianjin Aopusheng Chemical Reagents Co., Ltd. (Tianjin, China). Nutrient agar (NA), nutrient broth (NB), and bovine serum albumin (BSA, AR) were obtained from Zhuoyi Biotechnology Co., Ltd. (Shanghai, China). All chemicals and reagents used in this study were of analytical grade purity.

### 2.2. Preparation of Deep Eutectic Agent

The citric acid–deep eutectic agent (CA-DEA) was prepared by mixing CA and ChCl at different molar ratios ranging from 1:1 to 4:1. The mixtures were heated from 60–100 °C under continuous stirring until a clear, homogeneous liquid was obtained, following a previously reported optimized preparation procedure [9]. According to the results of that study, the 4:1 CA-DEA formulation demonstrated the most favorable physicochemical stability as well as the strongest antioxidant and antibacterial activities, and was therefore selected for subsequent application experiments in this study. To determine the appropriate incorporation level for shrimp preservation, a preliminary storage experiment was conducted using CA-DEA (4:1) solutions at concentrations of 1.25%, 2.5%, 5%, and 10%. Among these treatments, the 2.5% concentration demonstrated the most effective preservation performance and was therefore selected for subsequent coating preparation and storage experiments.

### 2.3. Preparation of Chitosan-DEA-Based Coating

Based on the preliminary findings, a CTS coating solution was prepared by dissolving 1% (*w*/*w*) CTS in 1.5% (*v*/*v*) acetic acid under continuous stirring at 50 °C until a clear and homogeneous solution was obtained. The optimized 2.5% CA-DEA (4:1; *w*/*w* of total solution) solution was then gradually added to the CTS solution, and the mixture was further heated at 50 °C for 30 min to obtain a uniform and stable CTS-CA-DEA composite coating. Fourier-transform infrared spectroscopy (FTIR, IR Affinity-1, Shimadzu, Kyoto, Japan) was performed within the spectral range of 4000–500 cm^−1^ to verify the successful formation of the CA-DEA system and its incorporation into the CTS matrix. Shifts in characteristic absorption peaks and changes in peak intensity were analyzed to evaluate intermolecular interactions (hydrogen-bond bonding) and confirm the structural compatibility of the composite coating.

#### 2.3.1. Antioxidant Properties of Coating

The antioxidant capacity of the coating solutions was evaluated using the DPPH and ABTS radical scavenging assays according to the method of Javed et al. [7], with slight modifications.

For the DPPH assay, a stock solution was prepared by dissolving 2 mg of DPPH in ethanol and adjusting the final volume to 50 mL, resulting in a DPPH concentration of approximately 0.1 mM. The solution was stored in the dark prior to use to prevent photodegradation. Equal volumes of 1% CTS, 2.5% CA-DEA (4:1), and their composite coating solution (1% CTS + 2.5% CA-DEA (4:1)) were mixed with the DPPH solution in a 1:1 (*v*/*v*) ratio. The mixtures were vortexed briefly to ensure complete mixing and then incubated in the dark for 30 min at room temperature. The absorbance was measured at 517 nm using a UV–visible spectrophotometer (Evolution 220, Thermo Fisher Scientific Inc., Waltham, MA, USA). The DPPH radical scavenging activity was calculated as follows:

(1) $$\mathrm{DPPH}\;\mathrm{scavenging}\;\mathrm{activity}\;(\%)=\left[1-\frac{\left(A_{i}-A_{j}\right)}{A_{0}}\right]\times 100$$

where

A_i_ represents the absorbance of the sample solution (sample + DPPH),

A_j_ represents the absorbance of the sample blank (ethanol + sample),

A_0_ represents the absorbance of the control (DPPH + ethanol).

An ABTS radical stock solution was prepared by dissolving 38.4 mg ABTS and 13.4 mg potassium persulfate in 10 mL of distilled water, followed by mixing equal volumes of both solutions and allowing the reaction to proceed for 12 h at room temperature in the dark to generate the ABTS radical cation (ABTS^+^). The resulting ABTS^+^ solution was diluted 20-fold with distilled water prior to use. Equal volumes of each coating solution were mixed with the diluted ABTS^+^ solution in a 1:1 (*v*/*v*) ratio and incubated for 6 min at room temperature. The absorbance was recorded at 734 nm using a UV–visible spectrophotometer (Evolution 201. 220. 260, Thermo Fisher Scientific Inc., Waltham, MA, USA). The ABTS radical scavenging activity was determined according to the equation:

(2) $$A\mathrm{BTS}\;\mathrm{scavenging}\;\mathrm{activity}\;(\%)=\left(1-\frac{A_{j}}{A_{0}}\right)\times 100$$

where

A_j_ represents the absorbance of the sample solution (ABTS + sample),

A_0_ represents the absorbance of the control (ABTS + water).

#### 2.3.2. Antibacterial Activity of Coating

The antibacterial activity of 1% CTS, 2.5% CA-DEA (4:1), and composite coating solution (1% CTS + 2.5% CA-DEA (4:1)) was evaluated against Gram-negative (*E. coli*; ATCC 44102) and Gram-positive (*S. aureus*; ATCC 26003) bacteria using the agar well diffusion method with slight modifications [16]. Fresh bacterial cultures were first cultivated in nutrient broth at 37 °C for 8–12 h to obtain actively growing cells. The bacterial suspensions were then uniformly spread onto nutrient agar plates. Sterile wells of 5 mm in diameter were aseptically punched into the agar medium, and 40 μL of each coating solution was carefully introduced into the wells. Cefixime (0.01 g dissolved in 40 mL of DMSO) served as a positive control, and DMSO alone as a negative control. Plates were incubated at 37 °C for 16–18 h, after which the inhibition zones were measured using an automated zone reader (ProtoCOL 3, Synbiosis, Cambridge, UK). All experiments were conducted in triplicate to ensure reproducibility.

### 2.4. Shrimp Preservation Experiment

Fresh *Penaeus chinensis* were purchased from a local aquatic market in Qinzhou, Guangxi, China, and immediately transported to the laboratory under refrigerated conditions for subsequent experiments. The preservation performance of the CTS-CA-DEA coating was evaluated under controlled refrigerated storage. Four treatment groups were established: (i) distilled water (control), (ii) 1% CTS solution, (iii) 2.5% CA-DEA (4:1) solution, and (iv) a composite coating containing 1% CTS + 2.5% CA-DEA (4:1). The shrimp samples were then immersed in the respective treatment solutions for 5 min, followed by air drying at room temperature for approximately 30 min to allow the coating to form on the shrimp surface. The treated samples were packaged in sterile polyethylene bags and stored at 4 ± 1 °C. For each treatment at each storage time (0, 3, 5, and 8 days), three independent replicates were analyzed, with six shrimp per replicate (18 shrimp per treatment per sampling day). Quality indicators were analyzed on days 0, 3, 5, and 8 to assess the preservation performance of each treatment group.

### 2.5. Physical Quality Parameters

#### 2.5.1. Color Measurement

Color variations in shrimp during refrigerated storage were quantified using a portable colorimeter (CR-400, Konica Minolta, Tokyo, Japan) according to the previously reported method with slight modifications [17]. Measurements were taken from the head, body, and tail regions, as well as from the peeled muscle tissue, to assess both external surface color and internal discoloration. The color coordinates L* (lightness), a* (redness/greenness), and b* (yellowness/blueness) were recorded using the CIE color system, providing an objective assessment of color variation and visual freshness during storage.

#### 2.5.2. Texture Profile Analysis (TPA)

Texture parameters were determined using a texture analyzer (TA-XT Plus, Stable Micro Systems, Godalming, Surrey, UK) operating in Texture Profile Analysis (TPA) mode, following the method described by Zhang et al. [18] with slight modifications. Measurements were performed on the second abdominal muscle segment of peeled shrimp using a cylindrical probe with a diameter of 2 mm. The instrument was operated under the following conditions: pre-test speed 1 mm/s, test speed 2 mm/s, and trigger force 5 N. The following texture parameters were recorded and calculated according to standard TPA definitions: hardness (maximum force required to compress the sample), cohesiveness (the ratio of the area under the second compression curve to the first, indicating internal bonding), springiness (the ability of the sample to recover its original shape after deformation), and adhesiveness (the work required to overcome the attractive forces between the sample and the probe). These parameters were used to assess structural integrity and the degree of firmness loss of shrimp muscle during storage.

#### 2.5.3. Dry Weight and Moisture Content Determination

Approximately 5 g of minced shrimp muscle was placed in a hot-air oven at 100 °C and dried until a constant weight was obtained, with mass measurements recorded at hourly intervals. Once weight equilibrium was achieved, the samples were further dried for an additional 30 min to ensure complete dehydration, and the final dry weight was recorded. The dry weight percentage was calculated using the following formula:

(3) $$\mathrm{Dry}\;\mathrm{weight}\;(\%)=\frac{W_{dry}}{W_{wet}}\times 100$$

Moisture content was determined based on the difference between the fresh and dry weights of the same samples, according to the method described by Peng et al. [9]. The moisture content (%) was calculated as:

(4) $$\mathrm{Moisture}\;\mathrm{content}\;(\%)=\frac{{W_{wet}-W}_{dry}}{W_{wet}}\times 100\;$$

where W_wet_ and W_dry_ represent the initial weight and final weight after drying, respectively. All analyses were performed in triplicate to ensure reproducibility, providing quantitative indicators of water loss and tissue dehydration during storage.

#### 2.5.4. pH

The pH of shrimp muscle was measured according to the method described by Echeverría et al. [19] with slight modifications. Approximately 1 g of fresh shrimp tissue was finely minced and homogenized with 5 mL of distilled water. The pH meter was calibrated using standard buffer solutions prior to measurement, and the pH value of the homogenized sample was then recorded in triplicate.

### 2.6. Biochemical Quality Parameters

#### 2.6.1. Protein Content

Protein content was analyzed according to the Chinese National Standard method (GB 5009.5-2016) [12,20], with minor adjustments. Approximately 1 g of homogenized shrimp muscle was digested with copper sulfate (0.4 g), potassium sulfate (6 g), and concentrated H_2_SO_4_ (20 mL) in a digestion tube at 420 °C until the solution became clear and colorless. After cooling, the digest was diluted with distilled water, made alkaline with sodium hydroxide, and the released ammonia was distilled and titrated with standardized hydrochloric acid. The total nitrogen content was calculated based on the titration results according to the standard Kjeldahl procedure. Crude protein content was then obtained by multiplying the total nitrogen by a conversion factor of 6.25, as recommended for general food matrices in GB 5009.5-2016, and expressed as g protein per 100 g of sample.

#### 2.6.2. Total Volatile Basic Nitrogen (TVB-N)

The TVB-N content of shrimp samples was determined following the Chinese National Food Safety Standard GB 5009.228–2016 [21] with slight modifications. Minced shrimp (2.0 g) was homogenized with 40 mL of distilled water, allowed to equilibrate for 30 min, and then filtered. A 10 mL aliquot of the filtrate was transferred into a distillation apparatus containing 10 mL of boric acid and five drops of mixed indicator (methyl red and bromocresol green, 1:5 *v*/*v*). Subsequently, 5 mL of magnesium oxide suspension was added, and the mixture was subjected to steam distillation for 5 min. The distillate was collected and titrated with 0.0100 mol/L HCl until a purple-red endpoint was reached. A reagent blank was processed simultaneously. All measurements were performed in triplicate, and results were expressed as mean values. The TVB-N content was calculated using the following equation:

(5) $$X=\frac{(V_{1}-V_{2})\times c\times 14}{m\times (V/V_{0})}\times 100$$

where;

*V*_1_ and *V*_2_ are the titration volumes of the sample and blank (mL), *c* is the molar concentration of HCl (mol/L), 14 is the nitrogen equivalent (mg/mmol), *m* is the sample mass (g), *V* is the aliquot volume (10 mL), and *V_0_* is the total extract volume (40 mL).

#### 2.6.3. Peroxide Value (PV)

PV was determined using a ferric thiocyanate colorimetric method based on Karlsdottir et al. [22], with slight modifications. Shrimp muscle (2.0 g) was mixed with 10 mL of *n*-hexane-ethanol (2:1, *v*/*v*) and centrifuged at 3000 rpm for 10 min. An aliquot of the supernatant lipid extract (0.3 mL) was then collected and reacted with 50 μL of 10 mM ammonium thiocyanate and 50 μL of 5 mM ferric chloride solution. The reaction mixture was incubated at room temperature for 5 min in the dark, and the absorbance was measured at 500 nm using a UV–visible spectrophotometer. A standard curve was prepared using serial dilutions of a 5 mM ferric chloride solution. The PV of each sample was calculated from the standard curve and expressed as meq O_2_/kg fat using the following equation:

(6) $$\mathrm{PV}\;(\mathrm{meq}\;O_{2}/\mathrm{kg}\;\mathrm{fat})\;=\;\frac{\mathrm{PV}\;\mathrm{standard}\;\mathrm{curve}\;\times \;\mathrm{Volume}\;\mathrm{of}\;\mathrm{extracted}\;\mathrm{lipids}}{\mathrm{Sample}\;\mathrm{weight}\;(g)}$$

### 2.7. Instrumental Sensory Evaluation

The aroma profile of fresh and stored shrimp samples was assessed using an NI400 electronic nose (iSensortalk-N series, Beijing iSensortalk Technology Co., Ltd., Beijing, China); as an objective sensory evaluation tool, following slight modifications of established protocols [23]. Minced shrimp (5 g) was transferred into a 20 mL airtight headspace vial and equilibrated at 40 °C for 15 min to promote the release of aroma compounds. After equilibration, the vial was connected to the sampling port of the E-nose instrument, and the headspace volatiles were automatically drawn into the sensor chamber under controlled flow conditions. Sensor signals were recorded for 120 s, including baseline stabilization, signal acquisition, and an automated chamber cleaning stage. The stabilized mean signal value for each sensor was extracted and used for subsequent multivariate statistical analysis of odor pattern changes during storage. All measurements were conducted in triplicate, and results are presented as mean values.

The E-nose used in this study contained 14 metal-oxide sensors, each designed to detect specific classes of volatile compounds commonly associated with seafood spoilage and freshness. These sensors exhibited sensitivity to volatile hydrides; a wide range of alcohols, aldehydes, and ketones; inorganic and organic sulfides; methylated and aromatic hydrocarbons; polycyclic aromatic hydrocarbons and ammonia; organic acids, ethers, and esters; nitrogen-containing volatiles; short-chain hydrocarbons; aromatic amines; macromolecular aromatic compounds; oxygenated organics; and long-chain alkanes and olefins.

### 2.8. Statistical Analysis

All experimental data were analyzed using two-way analysis of variance (ANOVA), with treatment and storage days as fixed factors, including their interaction effect (treatment × storage time), followed by Tukey’s HSD test (α = 0.05). Prior to ANOVA, the assumptions of normality and homogeneity of variance were evaluated using Shapiro–Wilk and Levene’s tests, respectively. The same statistical approach was applied to all measured variables. For color and texture analyses, individual shrimp were used as replicates (*n* = 18 per treatment per storage day), whereas for other analyses, six shrimp were pooled to form one replicate, resulting in three independent replicates per treatment per storage day (*n* = 3). All analyses were conducted in SPSS version 26.0 (IBM Corp., Armonk, NY, USA), and results are reported as mean ± SD.

## 3. Results and Discussion

### 3.1. Characterization and Properties of Chitosan Coating

#### 3.1.1. FTIR Analysis

**Figure 1:**
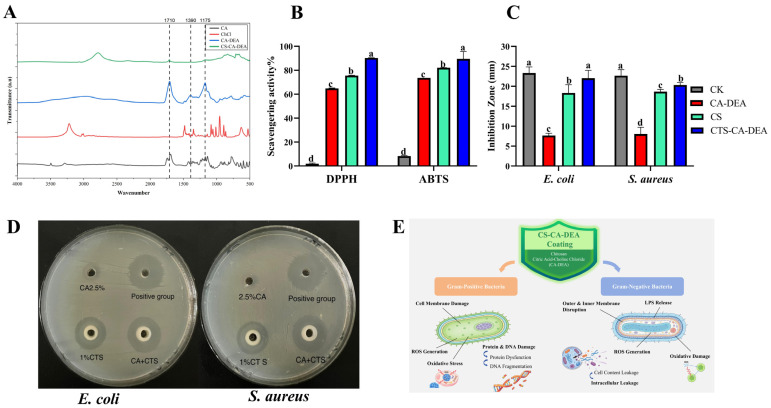
Characterization and functional properties of coating formulations: (**A**) FTIR spectra of CA, ChCl, CA-DEA, and CTS-CA-DEA; (**B**) antioxidant activity (DPPH and ABTS assays); (**C**) antibacterial activity against test bacteria; (**D**) representative inhibition zones; and (**E**) schematic illustration of the antibacterial mechanism of the CTS-CA-DEA coating. Groups with different letters are significantly different from each other (*p* < 0.05).

The FTIR spectra of CA, ChCl, CA-DEA, and the CTS-CA-DEA composite coating are presented in Figure 1A, providing clear evidence of eutectic formation and successful incorporation into the CTS matrix. Pure CA exhibited a characteristic carbonyl (C=O) stretching band at approximately 1710 cm^−1^, along with C–O stretching vibrations near 1175 cm^−1^, while ChCl displayed typical absorption bands associated with C–N and C–O stretching vibrations in the 1200–1000 cm^−1^ region. In the CA-DEA spectrum, peak broadening and intensity changes around 1710, 1390, and 1175 cm^−1^ indicate the formation of a hydrogen-bonded interaction system between CA and ChCl. After incorporation into CTS, these characteristic CA-DEA peaks remained detectable. Still, they showed attenuated rather than complete suppression, which can be attributed to the relatively low CA-DEA loading (2.5%) in the composite coating. Nevertheless, slight peak broadening and intensity reduction were observed, suggesting the formation of intermolecular interaction (primarily hydrogen bonding) between CA-DEA and the amino (–NH_2_) and hydroxyl (–OH) functional groups of CTS. These spectral features confirm the successful integration of CA-DEA into the CTS coating while maintaining the fundamental polymer backbone structure, thereby forming a compatible composite system capable of maintaining the desired bioactive functionality.

#### 3.1.2. Antioxidant Activity of Chitosan Coating

The antioxidant activities of the different formulations, evaluated by DPPH and ABTS radical scavenging assays (Figure 1B), showed clear and significant differences among treatments (*p* < 0.05). The CTS–CA-DEA composite coating exhibited the highest radical scavenging activity compared with CTS and CA-DEA alone, indicating a strong synergistic antioxidant effect. CTS contains abundant amino and hydroxyl groups capable of donating protons or electrons to neutralize free radicals [24,25]. At the same time, CA provides multiple carboxyl groups that can stabilize radical intermediates and chelate pro-oxidative metal ions [26,27]. When incorporated into the eutectic system, the hydrogen-bond interaction between CA and ChCl may enhance the availability and stability of these functional groups, thereby improving radical scavenging efficiency [7,9]. Furthermore, previous studies have demonstrated that eutectic systems can improve the dispersion and retention of antioxidant functionality within biopolymer matrices, resulting in significantly higher antioxidant performance than that of the individual components alone [10]. In comparison, the CTS–CA-DEA coating in the present study achieved markedly higher scavenging activity, highlighting the effectiveness of the eutectic-assisted composite system. This improvement can be attributed to the combined effects of enhanced intermolecular interactions and improved dispersion of antioxidant components within the chitosan matrix, which collectively enhance radical quenching capacity. Such synergistic antioxidant behavior is particularly beneficial for seafood preservation, as it helps suppress lipid oxidation and delays quality deterioration during refrigerated shrimp storage.

#### 3.1.3. Antibacterial Activity of Chitosan Coating

The antibacterial activity of the different formulations against Gram-positive and Gram-negative bacteria is presented visually in Figure 1D and quantitatively in Figure 1C, revealing clear formulation-dependent differences in inhibition performance. The antibiotic reference control produced the largest inhibition zones, reaching approximately 23–24 mm for both bacterial strains, which confirms the reliability of the experimental method. The 2.5% CA-DEA treatment exhibited relatively weak antibacterial activity, producing inhibition zones of about 7–8 mm, indicating limited effectiveness when applied alone. In comparison, 1% CTS showed moderate antibacterial performance, producing inhibition zones of approximately 18–19 mm, which is consistent with the well-documented antimicrobial mechanism of CTS involving electrostatic interactions between protonated amino groups and negatively charged bacterial cell membranes, leading to membrane disruption and increased permeability [13,14].

Notably, the CTS-CA-DEA composite coating demonstrated significantly enhanced antibacterial activity, with inhibition zones of approximately 21–22 mm against both Gram-positive and Gram-negative bacteria, significantly higher than those of CA-DEA and CTS alone (*p* < 0.05). Although the inhibition zones were slightly smaller than those of the antibiotic control, the CTS-CA-DEA coating exhibited substantially stronger antibacterial performance than the individual components, indicating a clear synergistic antibacterial effect. This enhancement may be attributed to a complementary mechanism, where CTS-induced membrane disruption facilitates the penetration of CA-DEA molecules into bacterial cells, while the acidic and hydrogen-bond-rich microenvironment generated by CA–DEA further interferes with bacterial metabolic processes and structural integrity [7,9]. Similar synergistic antibacterial enhancement has been reported in chitosan-based composite coatings containing bioactive additives for seafood preservation [15]. Consequently, the strong and broad-spectrum inhibition observed for the CTS-CA-DEA coating highlights its considerable potential to inhibit microbial proliferation and delay spoilage during refrigerated shrimp storage.

### 3.2. Effect of Chitosan Coatings on Physical Quality of Shrimp

#### 3.2.1. Color (L*, a*, and b*) Changes

**Figure 2:**
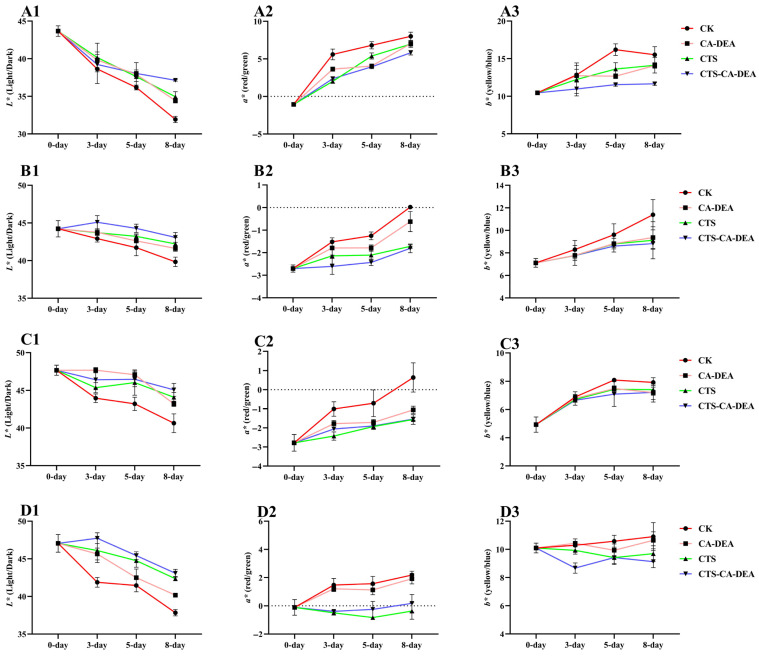
Changes in color parameters of shrimp during refrigerated storage measured at different anatomical regions: head (**A1**–**A3**), body with carapace (**B1**–**B3**), body without carapace (**C1**–**C3**), and tail (**D1**–**D3**). Panels represent L*, a*, and b* values, respectively.

Color is a critical freshness indicator for shrimp and is closely associated with enzymatic browning, melanosis development, pigment oxidation, and microbial spoilage during refrigerated storage [28]. The changes in the shrimp color parameters (L*, a*, and b*) during refrigerated storage are shown in Figure 2(A1–D3). Across all anatomical regions, progressive discoloration occurred as storage time increased [28]; however, the extent of these changes varied significantly depending on the treatment applied. The decline in visual quality was most pronounced in the control group, which exhibited a sharp reduction in L* values accompanied by marked increases in a* and b* values by day 8. These changes indicate rapid surface darkening, redness development, and yellowing, reflecting accelerated melanosis formation, pigment oxidation, microbial activity, and protein degradation [29]. Such deterioration patterns are characteristic of untreated shrimp during cold storage and are consistent with previous studies reporting uncontrolled oxidative and enzymatic processes in the absence of protective treatments [28,30]. In contrast, all coated samples showed significantly attenuated color changes, demonstrating that surface coatings effectively slowed the biochemical pathways responsible for shrimp discoloration.

Among the treatments, the CTS-CA-DEA composite coating provided the most effective color stabilization, consistently maintaining higher L* values while suppressing the increases in both a* and b* throughout storage. This effect was particularly evident in the tail region and peeled muscle tissue, where CTS-CA-DEA treated shrimp retained negative a* values and exhibited only minimal yellowing by day 8, indicating superior preservation of intrinsic muscle freshness. These findings closely align with our previous fish preservation study, in which CA-DEA-based coatings enhanced lightness, stabilized redness, and modulated yellowness [9] by limiting oxidative and microbial deterioration [31,32]. The improved color stability observed in shrimp can be attributed to combined barrier and antioxidant effects whereby the CTS matrix forms a semi-permeable barrier that restricts oxygen diffusion and suppresses microbial growth, while CA-DEA contributes antioxidant and metal-chelating functions that help stabilize pigments and lipids, and inhibit enzymatic browning. Collectively, these results confirm that the CTS-CA-DEA composite coating provides a significant improvement in color stability during refrigerated storage, reinforcing its potential suitability as a multifunctional and eco-friendly preservation strategy for shrimp quality during refrigerated storage.

#### 3.2.2. Visual Appearance Evaluation

**Figure 3:**
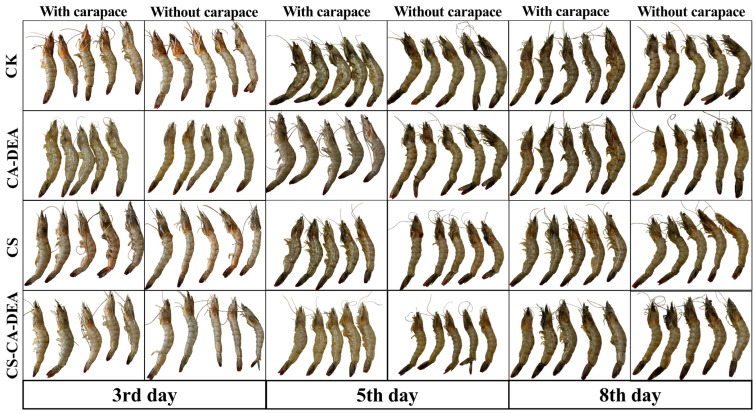
Visual appearance of shrimp treated with different coatings during refrigerated storage at 4 °C. Treatments include control (T1), CTS (T2), CA-DEA (T3), and CTS-CA-DEA (T4) observed at days 0, 3, 5, and 8. Images show shrimp with carapace and peeled shrimp muscle.

The visual observations of shrimp during refrigerated storage were consistent with the instrumental color measurement (L*, a*, b*) presented in Section 3.2.1. Control samples exhibited early surface darkening and uneven coloration by day 3, which progressively developed into pronounced melanosis, yellowing, and dull surface appearance by days 5–8, reflecting accelerated pigment oxidation and spoilage processes. Shrimp treated with CTS or CA-DEA individually showed partial mitigation of these visual changes, although some dark spots, dehydration, and loss of brightness were still observed during storage. In contrast, the shrimp coated with the CTS-CA-DEA composite maintained a bright and uniform appearance with minimal melanosis formation and better structural integrity throughout storage (Figure 3). These observations correspond well with the higher L* values and restrained changes in a* and b* parameters observed instrumentally, confirming that the CTS-CA-DEA coating effectively suppresses melanosis development and oxidative discoloration, thereby extending the visual shelf life and maintaining the commercial quality of shrimp under refrigeration.

#### 3.2.3. Moisture Loss & Dry Weight

**Figure 4:**
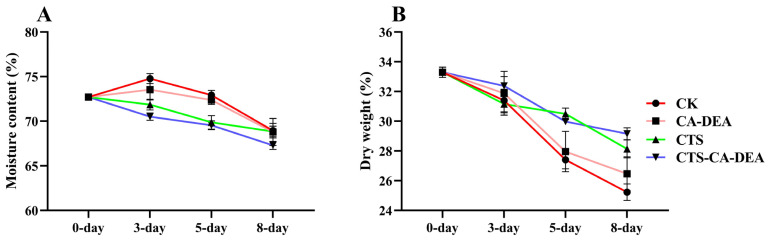
Effect of coating on shrimp moisture content (**A**) and dry weight (**B**) during storage.

Moisture loss and dry weight reduction are critical quality indicators that strongly influence the texture and sensory attributes of shrimp during refrigerated storage (Figure 4). As storage time progressed, the control samples exhibited the most pronounced moisture loss and a corresponding reduction in dry weight, reflecting the absence of a protective surface barrier and accelerated degradation of muscle structure. In contrast, coated shrimp samples showed significantly slower moisture loss (Figure 4A) and greater retention of dry matter (Figure 4B), indicating improved water-holding capacity and structural stability. CTS-based coatings are known to delay moisture loss by forming a semi-permeable barrier that limits water diffusion, thereby retarding dehydration of the underlying muscle tissue [33]. Notably, incorporation of CA-DEA further strengthened this protective effect by improving the functional properties of the coating, including its barrier properties and antioxidative capacity, which help to limit water evaporation, suppress oxidative degradation, and preserve structural components within the muscle matrix [9,34]. Consequently, shrimp treated with the CTS-CA-DEA composite coating maintained the highest moisture content and dry weight throughout storage, confirming that CA-DEA incorporation synergistically improved the barrier performance of the coating, thereby effectively delaying quality deterioration under refrigerated conditions.

#### 3.2.4. Texture Properties

Texture profile analysis demonstrated pronounced storage- and treatment-dependent changes in shrimp muscle quality (Table 1). Both hardness parameters increased progressively during refrigerated storage, with the control sample exhibiting the greatest increase by day 8, coinciding with the highest moisture loss and dry weight reduction. This excessive hardening is attributed to dehydration-induced protein aggregation and structural compaction of the muscle matrix [35], phenomena widely reported in shrimp muscle during refrigerated storage due to reduced water-holding capacity and myofibrillar protein denaturation [36]. In contrast, coated samples (particularly CTS and CTS-CA-DEA) maintained significantly lower hardness values, consistent with their improved moisture retention and preservation of dry matter (Figure 4). Cohesiveness and springiness exhibited relatively moderate changes in coated samples compared with the control, indicating better preservation of myofibrillar protein integrity and elastic recovery capacity, likely due to higher water-holding capacity [28]. Adhesiveness decreased sharply during early storage in all samples due to surface dehydration, followed by a partial increase in the control sample on day 8, which corresponded with increased drip loss and exudate formation. Coated samples, especially CTS-CA-DEA, maintained significantly lower adhesiveness throughout storage, aligning with their higher moisture content and dry weight. Overall, the strong concordance between texture parameters, moisture retention, and dry matter stability confirms that the CTS-CA-DEA composite coating effectively mitigated dehydration and helped maintain muscle structural integrity, thereby preserving the structural and textural quality of shrimp during refrigerated storage.

### 3.3. Effect of Chitosan Coatings on Biochemical Quality of Shrimp

#### 3.3.1. pH

**Figure 5:**
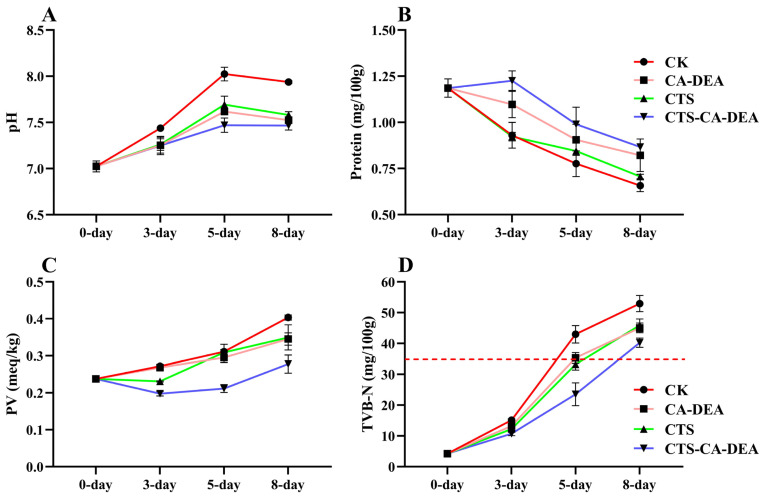
Changes in biochemical quality parameters of shrimp during refrigerated storage: (**A**) pH, (**B**) protein content (%), (**C**) peroxide value (PV, meq O_2_/kg fat), and (**D**) total volatile basic nitrogen (TVB-N, mg/100 g).

pH is a widely recognized indicator of spoilage in shrimp, reflecting the progression of biochemical and microbial degradation during storage. As shown in Figure 5A, the pH of the control sample increased rapidly from approximately 7.0 at day 0 to values exceeding 8.0 by day 5, surpassing the acceptability limit of 7.6 for shrimp and indicating accelerated spoilage. This pronounced alkalization is primarily attributed to autolytic activity and microbial enzymatic degradation of proteins, leading to the accumulation of volatile basic nitrogenous compounds such as ammonia and amines [37]. In contrast, shrimp treated with coatings exhibited significantly slower pH increases throughout storage. The reduced pH elevation in coated samples can be partly explained by the acidic nature of the CTS-based coating system, which has been reported to delay the pH rise in shrimp during refrigerated storage [17,38]. Moreover, the incorporation of CA-DEA further enhanced the preservation effect by strengthening the antimicrobial and antioxidant properties of the coating, thereby more effectively suppressing microbial metabolism and endogenous proteolytic activity. Under acidic conditions, protonation of amino groups enhances electrostatic interactions with negatively charged microbial cell membranes, leading to membrane disruption and inhibition of microbial growth [25]. Consequently, samples treated with the CTS-CA-DEA composite maintained lower pH values throughout storage, indicating that the incorporation of CA-DEA significantly delayed spoilage reactions and contributed to extending the refrigerated shelf life of shrimp.

#### 3.3.2. Protein Content

Protein is a key structural and nutritional component of shrimp muscle, and its degradation during refrigerated storage directly affects texture, water-holding capacity, and overall quality [39]. The protein content of shrimp decreased progressively throughout storage in all treatments (Figure 5B), with the control sample exhibiting the most pronounced decline, indicating extensive proteolysis and leaching of water-soluble proteins. In contrast, coated samples retained significantly higher protein levels, particularly the CTS-CA-DEA treatment, which consistently showed the slowest rate of protein loss. This improved protein stability can be attributed to the barrier properties of the coating, which reduce moisture loss and exudate formation, thereby limiting protein leaching. Additionally, the enhanced antioxidant and antimicrobial effects provided by CA-DEA incorporation help suppress oxidative protein degradation and microbial enzymatic activity. The strong correspondence between protein retention, higher moisture content, and moderated texture changes indicates that preservation of muscle proteins contributes to maintaining structural integrity and overall quality of shrimp during refrigerated storage.

#### 3.3.3. Peroxide Value

Peroxide value (PV), an indicator of primary lipid oxidation, increased progressively in all shrimp samples during refrigerated storage, reflecting the continuous formation of lipid hydroperoxides (Figure 5C). The control treatment exhibited the most rapid increase in PV, reaching the highest value by day 8, indicating greater susceptibility to oxidative deterioration. This trend is closely associated with the activity of psychrotrophic spoilage microorganisms, particularly lipase- and phospholipase-producing bacteria, which promote the release of free fatty acids that are highly prone to oxidation [40]. In contrast, coated samples showed significantly slower PV development, with the CTS-CA-DEA treatment consistently exhibiting the lowest values throughout storage. The restrained formation of hydroperoxides in treated shrimp can be attributed to the oxygen-barrier properties of CTS-based coatings, which limit oxygen diffusion to the muscle tissue and slow lipid oxidation reactions [17,41]. Moreover, the incorporation of CA-DEA likely enhanced oxidative stability by providing additional antioxidant functionality and improving the intermolecular interactions within the coating matrix, thereby further reducing permeability to oxygen and pro-oxidant species.

#### 3.3.4. Total Volatile Basic Nitrogen

Total volatile basic nitrogen (TVB-N), a widely accepted indicator of seafood spoilage, reflects the accumulation of alkaline nitrogenous compounds such as ammonia and amines formed mainly through microbial degradation of nitrogen-containing constituents [42]. As shown in Figure 5D, TVB-N values increased progressively in all shrimp samples during refrigerated storage, with the control (T1) exhibiting a rapid and pronounced rise, reaching the highest value by day 8. This sharp increase is attributed to the combined action of spoilage bacteria and endogenous enzymes, which intensify protein and non-protein nitrogen breakdown during storage [43]. The TVB-N content of the control exceeded the commonly accepted spoilage threshold of 30–35 mg/100 g for marine products by day 5, indicating a loss of freshness [44]. In contrast, coated samples, particularly those incorporated with CA-DEA, showed significantly slower TVB-N accumulation and remained below or close to the acceptability limit during the early and mid-storage periods. However, by day 8, even the CTS-CA-DEA-treated shrimp exceeded the threshold value, indicating the progression of spoilage at extended storage time. Based on these observations, the acceptable storage period in this study was estimated according to the TVB-N acceptability threshold. Accordingly, the CTS–CA-DEA treatment prolonged the estimated acceptable refrigerated storage period to approximately 7 days. In contrast, the uncoated control samples exceeded the acceptability threshold before 5 days, indicating rapid spoilage in the absence of protective treatment. This comparison confirms that the CTS-CA-DEA coating effectively extended the acceptable storage period by approximately 3 days, highlighting its significant role in delaying microbial and biochemical deterioration. The delayed formation of TVB-N in treated shrimp can be attributed to the antimicrobial effectiveness of CTS-based coatings, which restrict microbial proliferation and reduce enzymatic deamination reactions [17,40]. In addition, although the CTS–CA-DEA system exhibited enhanced antibacterial activity in vitro (Figure 1C), this result is interpreted as supporting evidence of potential spoilage inhibition rather than a direct measure of microbial dynamics on shrimp during storage. Importantly, this delayed TVB-N accumulation is consistent with the observed slower pH increase (Section 3.3.1) and reduced lipid oxidation (PV; Section 3.3.3), confirming that the CTS–CA-DES coating effectively mitigates multiple spoilage pathways during refrigerated storage. Similar inhibitory effects on TVB-N development have been reported for CTS coatings applied to shrimp, confirming their effectiveness in retarding spoilage and extending refrigerated shelf life [17,30].

### 3.4. Effect of Chitosan Coatings on Sensory Data

**Figure 6:**
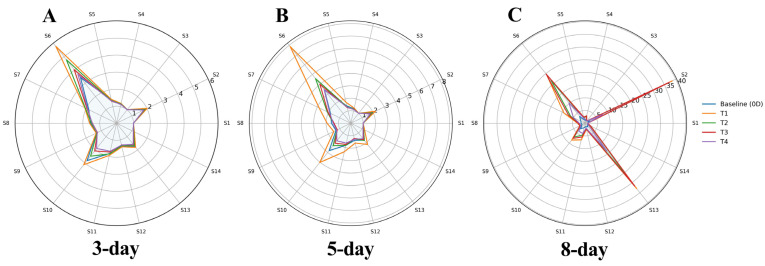
Effect of coating on shrimp sensory profiles during storage at 3 days (**A**), 5 days (**B**), and 8 days (**C**) measured using an electronic nose. Sensor responses: S1, volatile hydrides; S2, broad spectrum alcohols, aldehydes, ketones; S3, broad spectrum sulfides; S4, methyl aromatic hydrocarbons; S5, polycyclic aromatic hydrocarbons and ammonia; S6, broad spectrum alcohols, ethers, acids; S7, broad spectrum esters and nitrogen compounds; S8, short chain alkanes and olefins; S9, aromatic ammonia compounds; S10, nitrogenous compounds; S11, broad spectrum organic compounds; S12, methyl aromatic hydrocarbons; S13, volatile sulfides; S14, aromatic macromolecular compounds.

Electronic nose analysis revealed pronounced alterations in the volatile fingerprint of shrimp during refrigerated storage, reflecting progressive biochemical spoilage. As shown in the radar plots (Figure 6), sensors associated with lipid oxidation products, notably Sensor 2 (broad-spectrum alcohols, aldehydes, and ketones), Sensor 6 (alcohols, ethers, and acids), and Sensors 4 and 12 (methyl aromatic hydrocarbons), exhibited progressively higher responses with increasing storage time, particularly by day 8. Aldehydes, ketones, short-chain alcohols, and organic acids are well-known secondary lipid oxidation products formed through the degradation of polyunsaturated fatty acids in seafood, contributing directly to rancid off-odors [45,46]. In parallel, sensors linked to protein degradation and microbial metabolism, including Sensors 3 and 13 (inorganic and organic volatile sulfides), Sensor 9 (aromatic ammonia compounds), Sensor 10 (nitrogenous compounds), and Sensor 7 (esters and nitrogen compounds), showed marked signal enhancement during late storage. These volatile sulfur compounds and amines arise primarily from amino acid deamination, decarboxylation, and sulfur-containing amino acid breakdown, processes strongly associated with bacterial spoilage in crustaceans [47,48].

By day 8, the control samples exhibited the highest overall sensor intensities across both lipid- and protein-derived volatile groups, confirming advanced spoilage and aligning well with the elevated TVB-N and protein degradation observed in physicochemical analyses (Figure 5). In contrast, shrimp treated with the CTS-CA-DEA coating showed substantially attenuated responses in sensors associated with aldehydes, ketones, organic acids, ammonia, and volatile sulfides, indicating simultaneous suppression of lipid oxidation and proteolytic spoilage pathways. This reduction can be attributed to the combined antioxidant and antimicrobial properties of CTS-based coatings (Figure 1B,C), which limit oxygen diffusion, inhibit spoilage microflora, and slow enzymatic degradation reactions [17,40]. Although the CTS–CA-DEA system exhibited enhanced antibacterial activity in vitro (Figure 1C,D), the E-nose results in this study reflect changes in volatile compounds rather than direct measurement of microbial populations. Similar E-nose-based discrimination of seafood freshness, with strong correlations between sensor responses and microbial or chemical spoilage indicators, has been widely reported, validating the effectiveness of E-nose systems as rapid, non-destructive tools for monitoring lipid oxidation and protein degradation during storage [49]. Importantly, the E-nose observations are consistent with the trends in TVB-N, pH, and peroxide value, providing complementary evidence of delayed spoilage progression in treated samples. Overall, the E-nose results confirm that the CTS-CA-DEA coating significantly retards volatile formation associated with both oxidative rancidity and protein decomposition, thereby extending shrimp freshness during refrigerated storage.

## 4. Conclusions

The study demonstrated that incorporation of a citric acid–choline chloride deep eutectic agent (CA-DEA) into a CTS matrix significantly enhances the preservation performance of edible coatings applied to *Penaeus chinensis* during storage. The CTS-CA-DEA composite coating showed improved antioxidant and antibacterial functionality compared with a coating containing individual components, which contributed to slower physicochemical deterioration, reduced lipid oxidation (PV), lower TVB-N accumulation, moderated pH increase, and improved maintenance of shrimp quality during storage. These results indicate that the composite coating can delay, but not completely prevent, spoilage under refrigerated conditions, extending the acceptable freshness period of shrimp to approximately 7 days, compared with about 4 days for the uncoated control, thereby demonstrating an approximate 3-day extension in shelf life and confirming the practical preservation advantage of the CTS-CA-DEA coating. However, the present findings were obtained under controlled laboratory conditions, and further studies are required to evaluate the long-term stability, processing feasibility, and performance of the coating system under commercial storage and distribution environments. Overall, this work provides a systematic demonstration of DES-modified chitosan coatings as multifunctional preservation systems, offering an environmentally compatible and effective strategy for enhancing quality retention and acceptable storage duration of perishable aquatic foods.

## Figures and Tables

**Table 1:** Effect of chitosan coating treatments on the texture quality of shrimp during storage.

Hardness 1	0 Day	3rd Day	5th Day	8th Day
CK	213.66 ± 7.03d	275.83 ± 28.82b	361.16 ± 35.99a	385.50 ± 22.42a
CA-DEA	213.66 ± 7.03d	259.66 ± 26.34bc	343.66 ± 17.68ab	351.66 ± 15.97a
CTS	213.66 ± 7.03d	233.00 ± 14.66c	324.16 ± 33.22b	332.66 ± 32.46b
CTS-CA-DEA	213.66 ± 7.03d	212.33 ± 30.16d	306.50 ± 31.90b	316.66 ± 22.42b
**Hardness 2**	**0 day**	**3rd day**	**5th day**	**8th day**
CK	170.00 ± 5.29d	246.33 ± 43.66b	280.00 ± 35.08a	294.00 ± 10.82a
CA-DEA	170.00 ± 5.29d	220.00 ± 27.31c	264.66 ± 28.74ab	266.00 ± 32.08ab
CTS	170.00 ± 5.29d	172.00 ± 20.13d	256.33 ± 35.10b	263.50 ± 34.53b
CTS-CA-DEA	170.00 ± 5.29d	182.33 ± 33.33cd	233.50 ± 10.82c	243.00 ± 54.23b
**Cohesiveness**	**0 day**	**3rd day**	**5th day**	**8th day**
CK	0.53 ± 0.07b	0.56 ± 0.07ab	0.57 ± 0.07b	0.66 ± 0.05a
CA-DEA	0.53 ± 0.07b	0.54 ± 0.08ab	0.55 ± 0.04b	0.58 ± 0.08ab
CTS	0.53 ± 0.07b	0.53 ± 0.24b	0.55 ± 0.06b	0.57 ± 0.03ab
CTS-CA-DEA	0.53 ± 0.07b	0.49 ± 0.06c	0.48 ± 0.07c	0.49 ± 0.14b
**Springiness**	**0 day**	**3rd day**	**5th day**	**8th day**
CK	1.76 ± 0.26b	1.76 ± 0.26b	2.00 ± 0.12ab	2.11 ± 0.17a
CA-DEA	1.76 ± 0.26b	1.78 ± 0.21b	2.01 ± 0.17ab	2.10 ± 0.12a
CTS	1.76 ± 0.26b	1.76 ± 0.65b	1.98 ± 0.32b	2.05 ± 0.25ab
CTS-CA-DEA	1.76 ± 0.26b	1.76 ± 0.05b	1.96 ± 0.05b	2.01 ± 0.17ab
**Adhesion**	**0 day**	**3rd day**	**5th day**	**8th day**
CK	0.46 ± 0.08a	0.15 ± 0.05b	0.16 ± 0.05b	0.26 ± 0.05a
CA-DEA	0.46 ± 0.08a	0.10 ± 0.00c	0.15 ± 0.05b	0.21 ± 0.04ab
CTS	0.46 ± 0.08a	0.10 ± 0.06c	0.15 ± 0.05b	0.16 ± 0.05b
CTS-CA-DEA	0.46 ± 0.08a	0.11 ± 0.04bc	0.11 ± 0.04b	0.15 ± 0.06b

CK (Control); CA-DEA (Citric acid–choline chloride deep eutectic agent); CTS (Chitosan); CTS-CA-DEA (Chitosan–citric acid–choline chloride deep eutectic agent). Values are expressed as mean ± SD (*n* = 3). Different letters within the same column indicate significant differences among treatments (*p* < 0.05).

## Data Availability

The original contributions presented in the study are included in the article/Supplementary Material, further inquiries can be directed to the corresponding authors.

## Supplementary Materials

The following supporting information can be downloaded at: https://www.mdpi.com/article/10.3390/foods15091601/s1, Figure S1: Optimization of CA–DEA formulations at different CA:ChCl molar ratios based on shrimp quality parameters during storage: (A) L*, (B) a*, (C) b*, (D) pH, (E) dry weight, and (F) moisture content measured during 6 days of storage.

## Abbreviations

The following abbreviations are used in this manuscript:

PV	Peroxide value
CA-DEA	citric acid–choline chloride deep eutectic agent
CTS	Chitosan
TVB-N	Total volatile basic nitrogen
