# Supplementary material for: Synergistic Antimicrobial Effects of Citric Acid-Based Deep Eutectic Bioactive Agents in Chitosan Coatings for Refrigerated Shrimp Preservation
Source: Foods. 2026 May 6;15(9):1601. doi: 10.3390/foods15091601 (PMC13164262; doi:10.3390/foods15091601)
Supplement: Supplementary file 1 [file foods-15-01601-s001.zip › foods-4192240-supplementary.pdf]

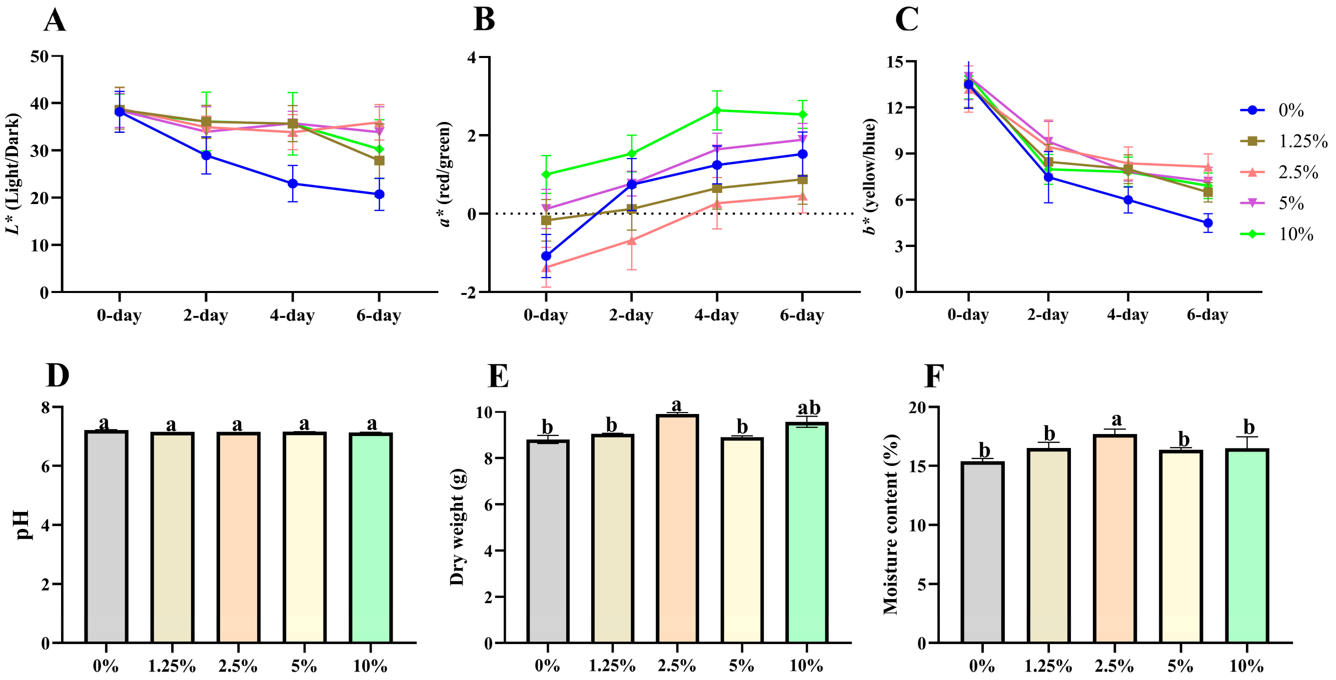


**Figure S1.** Optimization of CA–DEA formulations at different CA:ChCl molar ratios based on shrimp quality parameters during storage: (A) *L**, (B) *a**, (C) *b**, (D) pH, (E) dry weight, and (F) moisture content measured during 6 days of storage.
